# A Novel Pre-Kidney Transplant Risk Score to Optimize Waiting List Management

**DOI:** 10.3390/jcm15083045

**Published:** 2026-04-16

**Authors:** Lino Henkel, Katharina Könemann, Alison Kane, Göran R. Boeckel, Amélie F. Menke, Ana Harth, Philipp Houben, Hermann Pavenstädt, Wolfgang Arns, Stefan Reuter

**Affiliations:** 1 Department of Medicine D, University Hospital Münster, 48149 Münster, Germany; 2 Transplant Department, Merheim Medical Center, Cologne General Hospital, 51109 Cologne, Germany; 3 Department of General, Visceral and Transplant Surgery, University Hospital Münster, 48149 Münster, Germany

**Keywords:** kidney transplantation, renal transplantation, waitlist management, waiting list, comorbidity index, risk score, immunization

## Abstract

**Background**: Clinical tools to structure kidney transplant waitlist management at the time of listing are limited. We evaluated a simple, donor-independent clinical grading applied at waitlist registration to stratify patients according to post-transplant risk. **Methods**: We retrospectively analyzed 465 adult kidney transplant recipients from two German centers (2018–2023). Patients were assigned to three clinical grading groups based on age and comorbidities, and to three immunologic groups based on pre-immunization. One-year outcomes included mortality, graft loss, eGFR, albuminuria, and rejection. **Results**: Higher clinical grades were associated with worse one-year outcomes, including lower eGFR and higher rates of death or graft loss, whereas immunologic grading was associated with waiting time but not short-term post-transplant outcomes. These associations appeared robust to donor characteristics in sensitivity analyses. **Conclusions**: A simple, listing-time clinical grading may support structured waitlist management before donor information is available. External validation is required.

## 1. Introduction

Kidney transplantation (KTx) is the preferred treatment for end-stage renal disease (ESRD), demonstrating superior patient survival and quality of life outcomes in comparison to dialysis [1,2]. In Germany, the demand for kidneys significantly exceeds the supply, resulting in protracted waitlist times for potential donors [3]. Waitlisted patients frequently exhibit multiple comorbidities and frailty, necessitating continuous re-evaluation of eligibility by transplant centers to ensure the match between organ quality and recipient risk factors is optimal. Clinical guidelines recommend a comprehensive pre-listing evaluation that includes cardiac stress testing, vascular and pulmonary assessment, cancer screening, and infectious workup, in addition to individualized assessments. Of particular note, well-established risk factors such as cardiovascular disease, obesity, frailty, and psychosocial issues are thoroughly assessed for their impact on post-transplant outcomes [4,5,6]. Given the potential for a patient’s condition to deteriorate during dialysis, continuous reassessment of transplant suitability is recommended. Prognostic models have been developed for this heterogeneous group of patients to assess risk and probability of success prior to transplantation [7,8].

In light of the prevailing organ shortage and the aging population of end-stage renal disease (ESRD) patients, there is mounting pressure to enhance the efficacy of waitlist allocation and management [1,9,10,11]. However, standardized guidance for structured waitlist management remains limited. Existing recipient stratification approaches, such as comorbidity-based indices, functional assessments, and donor-dependent prediction tools, address related but distinct clinical questions and are often either more complex or not specifically designed for immediate use at waitlist registration (see Table 1) [12,13,14,15,16]. Consequently, a user-friendly clinical grading instrument was developed (based on the Charlson Comorbidity Index (CCI) and patient age) to stratify patients at listing. Additionally, an immunologic risk grade was delineated, incorporating preformed antibody levels and prior transplantation as pragmatic indicators of sensitization; other sensitizing events, such as pregnancy or blood transfusions, were not systematically available in this retrospective dataset. The hypothesis was developed that the combination of these gradings could enhance waitlist management by identifying patients with increased risk and directing customized follow-up and allocation strategies.

## 2. Materials and Methods

### 2.1. Study Design

We retrospectively analyzed 465 patients who underwent deceased-donor kidney transplantation at two German centers (Münster *n* = 308, Cologne *n* = 157) between 2018 and 2023. Patients receiving a graft from a living donor or under the age of 18 were excluded. Six patients who underwent two renal transplantations during the study period were included and counted as two cases. All available data were included (no exclusions for missing values), and records were anonymized prior to analysis. A formal a priori sample size calculation was not performed because the study aimed to develop and pragmatically validate a listing-time grading tool. In line with TRIPOD, we report outcome events and events-per-variable (EPV). The composite endpoint (all-cause mortality or graft loss) occurred in *E* = 60 patients; with *p* = 2 model parameters, EPV = 30, exceeding ≥10–20.

At the time of waitlist registration, subjects are assigned to one of three clinical risk groups based on the presence of comorbidities and age: Group I (low risk): ESRD with only “typical” comorbidities (e.g., renal anemia, secondary hyperparathyroidism, or hypertension) and no significant end-organ damage outside the kidneys. Group II (intermediate risk): ESRD plus one additional major comorbidity or systemic disease (e.g., diabetes mellitus, cardiovascular disease, or cancer). Group III (high risk): ESRD plus two or more major comorbidities or organ systems involved, or age ≥ 65 years. The patients were evaluated for the following comorbidities: diabetes mellitus (with end-organ damage), hypertension (with end-organ damage), coronary artery disease, heart failure or significant valvular heart disease, cerebrovascular disease, peripheral arterial disease, chronic lung disease, active malignancy, dementia, obesity (BMI > 30 kg/m^2^), and rheumatic disease. Clinical risk groups were defined a priori based on Charlson Comorbidity Index principles and clinical relevance, not data-driven selection. Additionally, patients were stratified into three immunologic risk groups based on preformed antibody level and transplant history: Group A (vPRA = 0%, no prior transplant), Group B (vPRA > 0% and ≤85%, or prior transplant), and Group C (vPRA > 85%). The immunologic grouping was defined a priori to reflect sensitization burden and transplant access. The vPRA threshold of 85% was chosen to identify marked sensitization, while prior transplantation was grouped with intermediate vPRA as a pragmatic marker of previous immunologic exposure. Other sensitizing events, such as pregnancy or blood transfusions, were not systematically available in this retrospective dataset. To facilitate a comparative analysis, we also evaluated established risk scores: The Karnofsky index [12], the Revised Cardiac Risk Score (RCRI) [13], the Myocardial Infarction Cardiac Arrest (MICA) [14], the CCI [15], and the modified Charlson comorbidity index for kidney transplantation (mCCI-KT) [16] in our cohort. For the Charlson Comorbidity Index, we defined scores of 1–2, 3–4, and ≥5 as separate groups, and for the CCI-KT, we defined scores of 0, 1, and ≥2 as separate groups. KDPI and KTOP were calculated per OPTN/Eurotransplant standards [17].

The study was approved by the local ethics committee (Ethik Kommission der Ärztekammer Westfalen-Lippe und der Medizinischen Fakultät der Universität Münster, No. 2024-302-f-S), which acted as the lead ethics committee for this retrospective dual-center study. Only anonymized data were transferred from the Cologne center.

### 2.2. Outcomes and Missing Variables

The primary outcomes were overall patient and graft survival. Patient survival was defined as time from transplant to death. Graft survival was defined as time to graft failure (return to dialysis or re-transplantation) or censoring at patient death with functioning graft. In addition, a composite endpoint of all-cause mortality or graft loss was analyzed as an exploratory summary outcome, whereas patient survival and graft-related outcomes were also assessed separately. Secondary outcomes included delayed graft function (dialysis within 7 days), biopsy-proven rejection (subdivided into acute or chronic humoral, borderline T-cell, T-cell, and mixed rejections on pathological examination), 1-year eGFR (CKD-EPI, mL/min/1.73 m^2^), and urine albumin-to-creatinine ratio (ACR, mg/g creatinine). This was followed by a moderation analysis to assess whether donor factors (donor age, number of mismatches, donor sex, and cold ischemia time) modify or attenuate the association between clinical grade and outcomes (robustness against donor characteristics).

One-year eGFR/uACR were unavailable when death or graft loss precluded 12-month sampling; these variables were not used to build or score the grade. Information on NT-days, biopsy-proven rejection episodes, and additional risk scores was collected only in Münster center (*n* = 308). Analyses including these variables were therefore restricted to patients from this center and are reported as such. For all other predictors with sporadic missing values (typically affecting ≤5% patients per variable), we used complete-case analysis in the respective models, excluding individuals with missing values for the variables involved. Given the very low proportion of missing data and the absence of evidence that missingness depended on the outcome, we did not perform multiple imputation.

### 2.3. Statistical Analysis

Continuous variables are reported as mean ± SD or median (IQR); categorical variables as *n* (%). We compared groups using Kruskal–Wallis tests (with post hoc Dunn-Bonferroni) or χ^2^ tests as appropriate. Effect sizes (Pearson’s r) are interpreted per Cohen’s criteria [18]. Correlations were assessed by Spearman’s rho. Survival curves were estimated by Kaplan–Meier and compared using the log-rank test. In addition, ordered differences across clinical grading categories were explored using a log-rank trend test, treating the clinical grades as an ordinal variable. Moderation analyses were performed using regression analysis with interaction terms using the PROCESS macro by Hayes [19]. Discriminative ability was assessed using ROC analysis with AUC and 95% confidence intervals (CIs) calculated via normal approximation. Statistical significance between paired AUCs was determined by non-overlapping CIs. Incremental value was assessed via nested binary logistic regression for the composite endpoint: Model 1 included donor risks (KDPI, KTOP); Model 2 added the Clinical Grading. Improvement was quantified by Δ-2 log likelihood (Likelihood Ratio Test) and change in Nagelkerke R^2^. A two-sided *p*-value < 0.05 was considered significant. Analyses were performed in SPSS 29 (IBM, Armonk, NY, USA).

## 3. Results

### 3.1. Study Population

**Figure 1:**
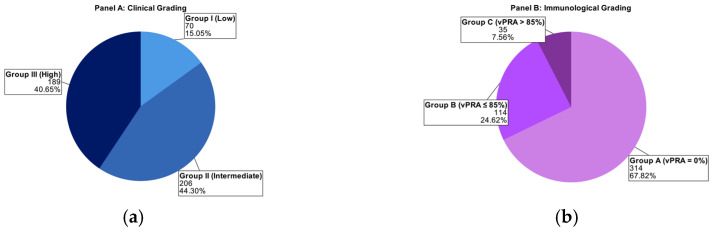
Distribution of study patients by clinical (**a**) and immunologic (**b**) grading at the time of waitlist registration. Clinical grading is based on age and comorbidity; immunologic grading is based on vPRA and transplant history. Most patients fell into intermediate or high clinical risk, while immunologic risk was low in the majority.

Table 2 summarizes the baseline characteristics. The mean age of the 465 transplant recipients was 56.7 ± 12.1 years; 63% were male, and the mean BMI was 26.3 ± 4.2 kg/m^2^. Before transplant, 83% of patients were on hemodialysis, 12.7% were on peritoneal dialysis, and 4.3% underwent preemptive transplantation. Overall, the mean time from the start of dialysis to transplantation was 2286.8 ± 1326 days. By allocation program, it was 2519 ± 1388 days for the Eurotransplant Kidney Allocation System (ETKAS, *n* = 343, 73.8%), 1543 ± 755 days for the European Senior Program (ESP, *n* = 110, 23.7%), and 2461 ± 1193 days for the Acceptable Mismatch Program (AM, *n* = 12, 2.6%) [20]. The mean time from the start of dialysis to being placed on the waiting list was 748 ± 883 days (range 0–5432 days). The mortality rate was 6%, and 12% of patients experienced graft failure. Delayed graft function occurred in 34.6% of recipients, and 16.9% (only Münster data available) had biopsy-proven rejection. Figure 1 shows patient distribution by clinical (Figure 1a) and immunologic (Figure 1b) risk groups.

### 3.2. Survival

**Figure 2:**
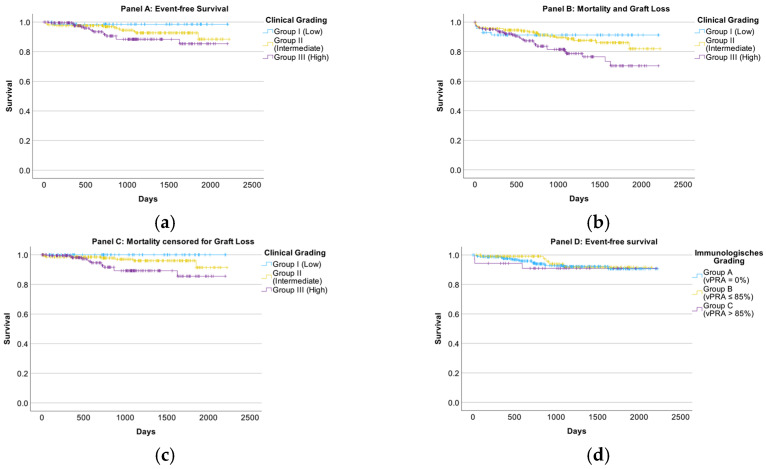
Kaplan–Meier of Event-free survival, categorized by clinical (**a**) and immunologic grading (**d**), mortality or graft loss, categorized by clinical grading (**b**), and mortality censored for graft loss, categorized by clinical grading (**c**). *n* = 465.

Figure 2 shows outcomes by risk group. Event-free survival did not differ significantly across clinical grades in the overall log-rank comparison (*p* = 0.073). However, when the clinical grades were analyzed as ordered categories, a significant trend across groups was observed (*p* = 0.022), suggesting a graded association that should be interpreted cautiously (Figure 2a). Furthermore, significant differences were observed when graft loss was included as an endpoint (*p* = 0.037) and when patient survival was censored for graft loss (*p* = 0.012) (Figure 2b,c). In contrast, stratification by immunologic grade showed no significant differences in survival (patient or event-free) at one year (all *p* > 0.15) (Figure 2d).

### 3.3. Graft Function

**Figure 3:**
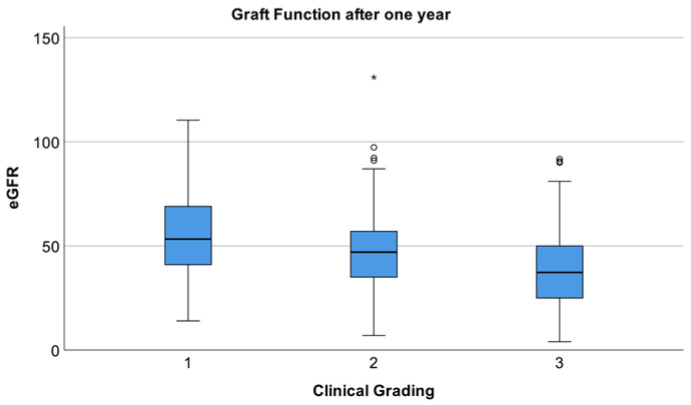
Box plot: Comparison of eGFR values at one year, categorized by clinical grade (1: *n* = 66, 2: *n* = 195, 3: *n* = 182). Circles indicate mild outliers (1.5–3 × IQR); stars (*) indicate extreme outliers (>3 × IQR).

The one-year graft function exhibited variability according to clinical grade, as illustrated in Figure 3. The mean eGFR (CKD-EPI) was 55.8 ± 21.7 mL/min/1.73 m^2^, 47.2 ± 18.2 mL/min/1.73 m^2^, and 39.1 ± 18.5 mL/min/1.73 m^2^ in groups I, II, and III, respectively (*p* < 0.001). Subsequent post hoc analyses substantiated discrepancies between all group pairs (Group I vs. II, *p* = 0.014; I vs. III, *p* < 0.001; II vs. III, *p* < 0.001). The median urine ACR was 29.5 (interquartile range [IQR] 16.8–76.1), 29.2 (15–65.8), and 39.2 (19.4–164.8) mg/g in groups I, II, and III, respectively. The overall *p*-value was 0.010, and a significant difference was observed between groups II and III (*p* = 0.009) (Supplementary Figure S1). Conversely, immunologic risk grouping exhibited no statistically significant disparities in eGFR (*p* = 0.308) or ACR (*p* = 0.899) (Supplementary Tables S1 and S2).

### 3.4. Rejection and Waitlist Times

The occurrence of biopsy-proven acute rejection exhibited no statistically significant differences across clinical grades (*p* = 0.977) or immunologic grades (*p* = 0.493), as illustrated in Supplementary Tables S3 and S4. Non-transplantable (NT) days, reflecting temporary ineligibility, had a median of 191 (IQR 0–538) but differences were not statistically significant overall (*p* = 0.069) or within the ETKAS group (*p* = 0.578) (see Supplementary Table S9). Group III patients exhibited reduced transplant waiting times on average (see Supplementary Table S5), a phenomenon that is presumably attributable to the ESP allocation. However, when the analysis was restricted to ETKAS patients, no correlation remained. As indicated by the statistical significance indicated in Supplementary Table S7 (*p* = 0.007), there was an observed correlation between higher clinical grade and longer evaluation-to-listing intervals. Detailed waitlist analyses are presented in Supplementary Tables S5–S10. There were also differences in waiting time between the immunological grading groups (see Supplementary Table S6) for all patients (*p* < 0.001) and ETKAS-only patients (*p* = 0.013), restricted to groups 1 and 2 (*p* < 0.001 and *p* = 0.018, respectively). When stratified by immunologic grade, NT days differed significantly (*p* < 0.001; Supplementary Table S10), whereas listing time did not (*p* = 0.919; Supplementary Table S8). By clinical grade, NT-day differences were not significant overall (*p* = 0.069; Supplementary Table S9).

### 3.5. Influence of Donor Characteristics

To rule out the influence of donor-related characteristics on the predictive accuracy of the clinical grading score, a moderation analysis was conducted on specific parameters, including donor age, donor sex, number of mismatches, and cold ischemia time. The analysis revealed significant correlation and association between donor age and eGFR (both *p* < 0.001) one year after transplantation, but the influence on the predictive power of the score could be ruled out given the non-significant interaction term (*p* = 0.281). However, the effect of clinical grading was no longer significant at the average donor age (*p* = 0.061), likely due to the study’s limited statistical power. Although donor characteristics (notably donor age) were associated with graft function in univariate models, they did not materially alter the prognostic contribution of clinical grade in interaction analyses (see Supplementary Tables S11 and S12).

### 3.6. Charlson Comorbidity Index

With respect to survival and survival plus graft loss (see Supplementary Figure S2), no statistically significant differences were observed between the CCI groups (*p* = 0.067 and 0.348, respectively). However, for CCI-KT, significant differences were identified between group 3 and group 1 or 2 (*p* = 0.04; *p* = 0.004, respectively). An examination of eGFR after one year (see Supplementary Table S13) reveals a statistically significant difference in scores among all groups (*p* < 0.001 and *p* = 0.005, respectively).

### 3.7. Karnofsky Index, RCRI, MICA

For the transplant recipients from the Münster center, the MICA, RCRI, and Karnofsky Index were also examined with regard to survival and graft function one year after transplantation (see Supplementary Figure S3 and Supplementary Tables S15 and S16). Significant differences in survival and GFR one year after kidney transplantation were found for the Karnofsky Index (*p* = 0.005 and *p* = 0.046, respectively) and the MICA score (*p* = 0.001 and *p* < 0.001, respectively) but not for RCRI (*p* = 0.282 and *p* = 0.298, respectively).

### 3.8. Score Head-to-Head Comparison

Finally, an exploratory head-to-head comparison of the scores was performed using ROC curve analysis for the composite endpoint of all-cause mortality or graft loss. All indices demonstrated modest discriminative ability for the composite endpoint (Supplementary Table S17 and Figure S4). The Clinical Grading Score achieved an AUC of 0.56 (95% CI 0.48–0.64) indicating limited-to-moderate discrimination, comparable to CCI-KT (AUC 0.56, 95% CI 0.48–0.65, *p* = 0.94) and MICA (AUC 0.59, 95% CI 0.50–0.68, *p* = 0.23), and superior to CCI (AUC 0.54, 95% CI 0.45–0.62, *p* = 0.52), RCRI (AUC 0.52, 95% CI 0.44–0.60, *p* = 0.41), and Karnofsky Index (AUC 0.41, 95% CI 0.33–0.48, *p* = 0.01).

In patients from both centers, the clinical grading score (AUC 0.57, 95% CI 0.5–0.64) was not significantly inferior to the established prognostic indices KDPI (AUC 0.59, 95% CI 0.5–0.67, *p* = 0.7) and KTOP (AUC 0.60, 95% CI 0.52–0.68, *p* = 0.46), despite not requiring donor data (Supplementary Table S18 and Figure S5). However, it showed no incremental prognostic value (Δ-2LL = 0.407, χ^2^(1) = 0.407, *p* = 0.52; ΔNagelkerke R^2^ = 0,02).

## 4. Discussion

We present a simple pre-transplant risk stratification tool using clinical parameters—age and comorbidity—alongside immunologic sensitization, both assessed at the time of waitlisting. Our clinical grading system was associated with higher rates of death or graft loss and one-year outcomes, including eGFR and albuminuria, while immunologic grading was associated with wait time but not with graft or patient outcomes. These findings indicate that clinical and immunologic dimensions provide complementary perspectives: clinical grade informs short-term post-transplant prognosis; immunologic burden primarily constrains access. Unlike KDPI/KTOP—requiring detailed donor–recipient information—and counseling tools such as iChoose Kidney or EPTS, our score is purpose-built for the listing time point, requires no calculations or donor data, and relies solely on routine clinical information. While KDPI has shown limited predictive value in European settings, and tools such as iChoose Kidney and EPTS aid in counseling or post-listing decisions, they are not suited for real-time risk stratification at the point of registration [21,22,23,24].

The novelty of the present study does not lie in showing that older or more comorbid patients have poorer post-transplant outcomes, as this is well established. Rather, the novelty lies in translating this routinely available information into a simple, donor-independent grading that can be applied at the time of listing and may support structured waitlist management in everyday clinical practice. Our approach offers a pragmatic advantage: it leverages readily available information without requiring complex calculations or real-time donor data. Importantly, the grading revealed a consistent trend in outcomes—patients in higher clinical grades experienced lower eGFR and higher albuminuria at one year, even when survival alone was not significantly different. This graded association supports its potential for practical use. We acknowledge that categorization of age and comorbidity may reduce predictive granularity compared with continuous-variable modeling. However, this was a deliberate design choice, as the primary aim of this study was to provide a simple, donor-independent grading system for use at the time of listing rather than to derive a maximally optimized multivariable prediction model. In this context, categorization improves interpretability and may facilitate implementation in routine clinical workflows.

Rejection rates did not differ significantly between clinical or immunologic groups, which aligns with the fact that variables directly influencing rejection-such as HLA mismatch, ABO incompatibility, and cold ischemia time-are not part of this scoring system. Moreover, this could also be related to rigorous exclusion of unacceptable HLA antigens [25]. The one-year follow-up may also be too short to detect differences, as the incidence of antibody-mediated rejection increases over time [26,27]. Nonetheless, higher-risk patients exhibited clearly worse graft function, suggesting that comorbidity-related resilience plays an independent role for organ function.

A notable and somewhat counterintuitive finding was that Group III patients (≥65 years or multiple comorbidities) had shorter average waiting times. This can be explained by their eligibility for the Eurotransplant Senior Program (ESP), which prioritizes allocation among older recipients and donors to minimize ischemic time and maximize organ utility [28]. This program effectively reduces waiting time for elderly patients, who are also more vulnerable to extended dialysis exposure [29]. However, in the standard ETKAS allocation system, clinical grade had no impact on waiting time, reinforcing that age/comorbidity are not currently integrated into matching criteria [30]. Importantly, our results highlight differences in time from dialysis initiation to listing. Higher clinical risk groups had significantly longer time-to-listing intervals, likely reflecting more complex evaluations and the need for medical optimization. Delays in listing can disproportionately harm elderly or comorbid patients, whose dialysis-associated mortality risk is elevated [9,29]. Streamlining listing processes for these patients—such as through parallel evaluation tracks or prioritizing early referrals—could mitigate this risk.

Immunologic grading, based on vPRA and previous transplant, did not predict one-year survival, graft loss, or rejection. However, it did correlate with prolonged waiting time, particularly for patients with vPRA > 85%. This is consistent with other findings from Eurotransplant and U.S. cohorts, which show that highly sensitized patients experience markedly reduced transplant opportunities—even in settings with dedicated programs [31,32]. For instance, Ferrante et al. report for Eurotransplant patients that transplantation rates fall precipitously for vPRA > 85% [31].

Although no correlation was observed between immunologic grade and time to listing, our findings support continued policy initiatives such as the Acceptable Mismatch Program (AM) and Mismatch Probability Point allocation to improve access for this vulnerable group [30,33]. In practice, clinical and immunologic stratification thus provide complementary information. Beyond its statistical associations, the proposed clinical grading may support routine waitlist management by helping define re-evaluation intervals, identify patients requiring intensified pre-transplant optimization, and structure communication within the multidisciplinary transplant team. As it does not require donor information, it can be applied directly at the time of listing and is intended to complement rather than replace existing predictive tools.

In center-specific analyses (Münster cohort), the Karnofsky Performance Status and MICA were associated with 1-year survival and/or eGFR, whereas RCRI showed no association. Classical comorbidity indices (CCI and CCI-KT) stratified graft function. Nevertheless, our clinical grading achieved comparable discrimination using only age and comorbidity at listing—without ICD coding, weighting schemes, or transplant-unspecific items—facilitating routine implementation at the time of registration. Functional indices like MICA and Karnofsky capture clinical complexity but suffer from variability in acquisition and inter-observer differences and showed non-monotonic group behavior in our cohort. In contrast, an age/comorbidity-based listing-time grade leverages universally available, low-error data, exhibits a dose–response relationship with outcomes, and directly informs operational decisions (re-evaluation intervals, prioritization of diagnostics; for operational examples see Supplementary Table S19) at the time of waitlist entry. These observations support population-specific validation rather than universal adoption of generic, non-contextual indices.

Donor age and number of mismatches associated with eGFR and uACR in univariate analyses, but did not attenuate the prognostic contribution of clinical grade in interaction models (Supplementary Tables S11 and S12), supporting robustness of the recipient-focused stratification. Nested logistic regression further confirmed this: while KDPI + KTOP dominated prediction of the composite endpoint, addition of the Clinical Grading Score yielded no significant improvement, reflecting saturation by donor risks. While donor indices optimize organ allocation, recipient comorbidity assessment using the simple Clinical Grading Score remains critical for pre-listing selection when donor data is unavailable.

From a broader clinical and policy perspective, such grading may also support more standardized care pathways across centers. For example, high clinical risk may warrant more frequent follow-up, early re-evaluation and prioritized optimization (e.g., frailty interventions, for operational examples see Supplementary Table S19). Integrating such a grading into national registry systems could support a more individualized and equitable waitlist strategy, especially in multi-center networks where resource allocation and medical complexity vary. It could also inform allocation policy design—similar to how the U.S. incorporates EPTS into kidney offer sequencing—though caution is needed to balance outcome optimization with fairness [33].

Ultimately, the strength of our proposed score lies in its ease of use and clinical relevance. It can be implemented with low burden and is compatible with existing data systems. Future efforts should aim to validate this score in larger and more diverse populations and to assess its impact on long-term outcomes, waitlist mobility, and patient satisfaction. Importantly, the present approach should be understood as a pragmatic, workflow-oriented tool for structured waitlist management at the time of listing, rather than as a replacement for established donor-dependent prediction models.

This study has several limitations. It is a retrospective, dual-center analysis with a modest sample size, introducing potential selection bias and incomplete data. Some predictors were only available in one center, which may limit the precision and generalizability of some of the subgroup analyses. Six patients received two transplants during follow-up and were counted twice, limiting statistical independence. The German patient cohort differs from international populations, with listing and waiting times that may not reflect broader Eurotransplant practices, reducing generalizability. The clinical grading system used broad comorbidity categories without standardized coding; future applications should align with ICD-10 for consistency. In addition, the categorized structure of the grading system may reduce predictive precision compared with continuously modeled variables, but was intentionally chosen to enhance simplicity and usability in routine clinical practice. Larger, prospective, multi-center studies are needed to validate the score and assess its impact on equitable transplant management. Furthermore, the one-year follow-up and absence of detailed HLA mismatch data, donor-specific antibodies, and other immunologic parameters restrict the interpretation of long-term graft outcomes and late immunologic injury, including antibody-mediated rejection. The present study should therefore be understood as addressing short-term post-transplant risk stratification rather than long-term prognostic modeling. A longer follow-up is needed to assess the value of this approach for long-term graft survival.

## 5. Conclusions

A simple, donor-independent clinical grading based on age and comorbidities may support structured waitlist management at the time of listing, for example, by helping define re-evaluation intervals and identify patients requiring intensified pre-transplant optimization. In contrast, the immunologic grading primarily reflected access-related aspects rather than short-term post-transplant outcome.

## Figures and Tables

**Table 1:** Comparison of recipient risk stratification approaches relevant to kidney transplant waitlist management.

Approach	Main Variables	Donor Variables Required	Applicable at Waitlist Registration	Complexity	Main Purpose
Charlson Comorbidity Index (CCI)	Comorbidities	No	Yes	Moderate	General comorbidity assessment
Karnofsky Performance Status	Functional Status	No	Yes	Low	Functional impairment/frailty surrogate
MICA/RCRI	Cardiac risk- related variables	No	Yes	Moderate	Perioperative cardiovascular risk
KDPI/KTOP	Donor and recipient variables	Yes	No	Moderate to high	Post-allocation outcome prediction
Clinical Grading (this study)	Age and comorbidities	No	Yes	Low	Structured waitlist management at listing

This comparison illustrates that the proposed Clinical Grading differs from established tools by focusing on immediate applicability at listing without requiring donor information or complex weighting schemes.

**Table 2:** Study Population.

Title 1	Title 2	Minimum	Maximum
Age at transplantation (years, mean ± SD)	56.65 ± 12.06	22	81
Sex (*n*, %)			
Female	172 (37%)		
Male	293 (63%)		
BMI (kg/m^2^, mean ± SD)	26.27 ± 4.23	12.7	39.02
Dialysis (*n*, %)			
hemodialysis	386 (83%)		
peritoneal dialysis	59 (12.7%)		
preemptive	20 (4.3%)		
Waiting time (days, mean ± SD)	2286.78 ± 1326.08	0	6748
Transplant program			
ETKAS	2519.07 ±1387.54		
ESP	1543.45 ± 755.35		
AM	2461.00 ± 1192.78		
Number of NT days (median, IQR) *	191 (0–537.75)	0	3720
Time from first renal replacement therapy to listing (days, mean ± SD)	748.27 ± 883.09	0	5432
Transplant program (*n*, %)			
ETKAS	343 (73.8%)		
ESP	110 (23.7%)		
AM	12 (2.6%)		
Renal disease (*n*, %)			
hypertension	71 (15.3%)		
diabetes	55 (11.8%)		
polycystic kidney disease	152 (32.7%)		
reflux nephropathy	10 (2.2%)		
glomerulonephritis or Alport-Syndrome	101 (21.7%)		
focal segmental sclerosis	7 (1.5%)		
interstitial nephritis	11 (2.4%)		
vasculitis	11 (2.4%)		
other	47 (10.1%)		
smoking (*n*, %)			
active	69 (14.8%)		
former	143 (30.8%)		
never	193 (41.5%)		
unknown	60 (12.9%)		
Patient survival (*n*, %)			
deceased	28 (6%)		
survived	437 (94%)		
Graft loss (*n*, %)			
yes	56 (12%)		
no	409 (88%)		
Delayed graft function (*n*, %)			
yes	163 (35.1%)		
no	296 (63.7%)		
unknown	6 (1.3%)		
Biopsy-proven rejections (*n*, %) *			
yes	52 (16.9%)		
no	256 (83.1%)		

* Only patients from Münster included (*n* = 308). Abbreviations: SD, standard deviation; BMI, body mass index; ETKAS, Eurotransplant Kidney Allocation System; ESP, Eurotransplant Senior Program; AM, Acceptable Mismatch Program; NT, not transplantable; IQR, interquartile range.

## Data Availability

The raw data supporting the conclusions of this article will be made available by the authors on request.

## Supplementary Materials

The following supporting information can be downloaded at: https://www.mdpi.com/article/10.3390/jcm15083045/s1, Figure S1: Box plot: Comparison of uACR values at one year categorized by clinical grade (1: *n* = 61, 2: *n* = 179, 3: *n* = 144).; Table S1: Mean eGFR (CKD EPI) stratified by immunologic grading.; Table S2: Mean uACR stratified by immunologic grading.; Table S3: Number of rejections within 1 year after renal transplantation stratified by clinical grading. Only Muenster patients (*n* = 308).; Table S4: Number of rejections within 1 year after renal transplantation stratified by immunologic grading. Only Muenster patients (*n* = 308).; Table S5: Mean waiting time in days stratified by clinical grading for all patients and patients receiving transplants through the ETKAS program (*n* = 465).; Table S6: Mean waiting time in days stratified by immunologic grading for all patients and patients receiving transplants through the ETKAS program (*n* = 465).; Table S7: Mean listing time (evaluation-to-listing interval) stratified by clinical grading. Missing values for 9 patients (*n* = 456).; Table S8: Mean listing time (evaluation-to-listing interval) in days stratified by immunologic grading. Missing values for 11 patients (*n* = 454).; Table S9: Number of NT days stratified by Clinical grading for all patients and patients receiving transplants through the ETKAS program. Only Münster patients (*n* = 308).; Table S10: Number of NT days stratified by Immunologic grading. Missing values for 2 patients. Only Münster patients (*n* = 306).; Table S11: Mortality, Mortality and Graft Loss, eGFR, uACR and Rejections stratified by Donor Characteristics regarding association and correlation (only eGFR, uACR, Rejections). *n* = 460 (missing data in 5 patients) for Mortality, Mortality + Graft Loss, eGFR and uACR. Only Muenster patients (*n* = 308) for Rejections.; Table S12: Mediator analysis of the interaction of donor age with clinical grading and eGFR 1 year after Transplantation. *n* = 460 (missing data in 5 patients).; Figure S2: Kaplan–Meier of Event-free survival categorized by CCI (Panel A) and CCI-KT (Panel C), mortality or graft loss categorized by CCI (Panel B) and CCI-KT (Panel D). *n* = 465.; Table S13: Mean eGFR (CKD EPI) stratified by Charlson Comorbidity Index and Charlson Comorbidity Index-KT; Table S14: Mean uACR stratified by Charlson Comorbidity Index and Charlson Comorbidity Index-KT.; Figure S3: Kaplan–Meier of Event-free survival categorized by Karnofsky Index (Panel A), MICA (Panel C) and RCRI (Panel E), mortality or graft loss categorized by Karnofsky Index (Panel B), MICA (Panel D) and RCRI (Panel F). *n* = 465.; Table S15: Mean eGFR (CKD EPI) stratified by Karnofsky Index, MICA (divided into 5 equal groups: <1.07%, <1.27%, <1.49%, <1.65% and ≥1.65%) and RCRI.; Table S16: Mean uACR stratified by Karnofsky Index, MICA (divided into 5 equal groups: <1.07%, <1.27%, <1.49%, <1.65% and ≥1.65%) and RCRI.; Figure S4: ROC curves of the composite endpoint (mortality or graft loss) from the Clinical Grading (orange), Charlson Comorbidity Index (CCI, yellow), CCI-KT (green), Karnofsky Index (purple), MICA (divided into 5 equal groups: <1.07%, <1.27%, <1.49%, <1.65% and ≥1.65%, blue) and RCRI (light purple). *n* = 308.; Table S17: Calculated AUC from ROC analysis (Figure S4) with 95% confidence intervals. Difference in AUC and statistical significance (p) between the Charlson Comorbidity Index (CCI), CCI-KT, Karnofsky Index, MICA (divided into 5 equal groups: <1.07%, <1.27%, <1.49%, <1.65% and ≥1.65%), RCRI to clinical grading determined by non-overlapping CIs. *n* = 308.; Figure S5: ROC curves of the composite endpoint (mortality or graft loss) from the Clinical Grading (orange), KDPI (only graft failure, yellow) and KTOP (after 1 year, purple). *n* = 308.; Table S18: Calculated AUC from ROC analysis (Figure S4) with 95% confidence intervals. Difference in AUC and statistical significance (*p*) between KTOP (1 year after RTx) and KDPI (only graft failure) to clinical grading determined by non-overlapping CIs. *n* = 308; Table S19. Real-world operational examples for each risk level according to clinical grading.

## Abbreviations

The following abbreviations are used in this manuscript:

ACR	Albumin-To-Creatinine Ratio
AM	Acceptable Mismatch Program
AUC	Area Under the Curve
BMI	Body Mass Index
CCI	Charlson Comorbidity Index
CKD-EPI	Chronic Kidney Disease Epidemiology Collaboration
eGFR	Estimated Glomerulary Filtration Rate
EPTS	Estimated Post Transplant Survival
ESP	Eurotransplant Senior Program
ESRD	End-Stage Renal Disease
ETKAS	Eurotransplant Kidney Allocation System
ICD	International Statistical Classification of Diseases and Related Health Problems
KDPI	Kidney Donor Profile Index
KTOP	Kidney Transplant Outcome Prediction
KTx	Kidney Transplantation
MICA	Myocardial Infarction Cardiac Arrest
mCCI-KT	Modified Charlson Comorbidity Index for Kidney Transplantation
NT	Not Transplantable
RCRI	Revised Cardiac Risk Index
ROC	Receiver Operating Characteristic
vPRA	Virtual Panel Reactive Antibodies

## References

[1] Chaudhry D., Chaudhry A., Peracha J., Sharif A. (2022). Survival for waitlisted kidney failure patients receiving transplantation versus remaining on waiting list: Systematic review and meta-analysis. BMJ.

[2] Dew M.A., Switzer G.E., Goycoolea J.M., Allen A.S., DiMartini A., Kormos R.L., Griffith B.P. (1997). Does transplantation produce quality of life benefits? A quantitative analysis of the literature. Transplantation.

[3] Deutsche Stiftung Organtransplantation (2023). Jahresbericht Organspende und Transplantation in Deutschland 2022. https://dso.de/SiteCollectionDocuments/DSO-Jahresbericht%202023.pdf.

[4] Quero M., Montero N., Rama I., Codina S., Couceiro C., Cruzado J.M. (2021). Obesity in Renal Transplantation. Nephron.

[5] McAdams-DeMarco M.A., Law A., King E., Orandi B., Salter M., Gupta N., Chow E., Alachkar N., Desai N., Varadhan R. (2015). Frailty and mortality in kidney transplant recipients. Am. J. Transplant..

[6] European Renal Best Practice Transplantation Guideline Development Group (2013). ERBP Guideline on the Management and Evaluation of the Kidney Donor and Recipient. Nephrol. Dial. Transplant..

[7] Moore J., He X., Liu X., Shabir S., Ball S., Cockwell P., Inston N., Little M.A., Johnston A., Borrows R. (2011). Mortality prediction after kidney transplantation: Comparative clinical use of 7 comorbidity indices. Exp. Clin. Transplant..

[8] Patzer R.E., Basu M., Larsen C.P., Pastan S.O., Mohan S., Patzer M., Konomos M., McClellan W.M., Lea J., Howard D. (2016). iChoose Kidney: A Clinical Decision Aid for Kidney Transplantation Versus Dialysis Treatment. Transplantation.

[9] Gill J.S., Tonelli M., Johnson N., Kiberd B., Landsberg D., Pereira B.J.G. (2005). The impact of waiting time and comorbid conditions on the survival benefit of kidney transplantation. Kidney Int..

[10] Singh P., Ng Y.-H., Unruh M. (2016). Kidney Transplantation Among the Elderly: Challenges and Opportunities to Improve Outcomes. Adv. Chronic Kidney Dis..

[11] Foreman K.J., Marquez N., Dolgert A., Fukutaki K., Fullman N., McGaughey M., Pletcher M.A., Smith A.E., Tang K., Yuan C.-W. (2018). Forecasting life expectancy, years of life lost, and all-cause and cause-specific mortality for 250 causes of death: Reference and alternative scenarios for 2016-40 for 195 countries and territories. Lancet.

[12] Péus D., Newcomb N., Hofer S. (2013). Appraisal of the Karnofsky Performance Status and proposal of a simple algorithmic system for its evaluation. BMC Med. Inform. Decis. Mak..

[13] Goldman L., Caldera D.L., Nussbaum S.R., Southwick F.S., Krogstad D., Murray B., Burke D.S., O’Malley T.A., Goroll A.H., Caplan C.H. (1977). Multifactorial index of cardiac risk in noncardiac surgical procedures. N. Engl. J. Med..

[14] Gupta P.K., Gupta H., Sundaram A., Kaushik M., Fang X., Miller W.J., Esterbrooks D.J., Hunter C.B., Pipinos I.I., Johanning J.M. (2011). Development and validation of a risk calculator for prediction of cardiac risk after surgery. Circulation.

[15] Charlson M.E., Pompei P., Ales K.L., MacKenzie C.R. (1987). A new method of classifying prognostic comorbidity in longitudinal studies: Development and validation. J. Chronic Dis..

[16] Park J.Y., Kim M.H., Bae E.J., Kim S., Kim D.K., Joo K.W., Kim Y.S., Lee J.P., Kim Y.H., Lim C.S. (2018). Comorbidities Can Predict Mortality of Kidney Transplant Recipients: Comparison with the Charlson Comorbidity Index. Transplant. Proc..

[17] Kidney Transplant Outcome Prediction Calculator. https://riskcalc.org/ktop/.

[18] Cohen J. (1992). A power primer. Psychol. Bull..

[19] Hayes A.F. (2015). An Index and Test of Linear Moderated Mediation. Multivar. Behav. Res..

[20] Eurotransplant Manual. https://www.eurotransplant.org/allocation/eurotransplant-manual/.

[21] Miller G., Ankerst D.P., Kattan M.W., Hüser N., Vogelaar S., Tieken I., Heemann U., Assfalg V. (2023). Kidney Transplantation Outcome Predictions (KTOP): A Risk Prediction Tool for Kidney Transplants from Brain-dead Deceased Donors Based on a Large European Cohort. Eur. Urol..

[22] Lehner L.J., Kleinsteuber A., Halleck F., Khadzhynov D., Schrezenmeier E., Duerr M., Eckardt K.-U., Budde K., Staeck O. (2018). Assessment of the Kidney Donor Profile Index in a European cohort. Nephrol. Dial. Transplant..

[23] Molnar M.Z., Nguyen D.V., Chen Y., Ravel V., Streja E., Krishnan M., Kovesdy C.P., Mehrotra R., Kalantar-Zadeh K. (2017). Predictive Score for Posttransplantation Outcomes. Transplantation.

[24] Dahmen M., Becker F., Pavenstädt H., Suwelack B., Schütte-Nütgen K., Reuter S. (2019). Validation of the Kidney Donor Profile Index (KDPI) to assess a deceased donor’s kidneys’ outcome in a European cohort. Sci. Rep..

[25] Ziemann M., Suwelack B., Banas B., Budde K., Einecke G., Hauser I., Heinemann F.M., Kauke T., Kelsch R., Koch M. (2022). Determination of unacceptable HLA antigen mismatches in kidney transplant recipients. HLA.

[26] Sellarés J., de Freitas D.G., Mengel M., Reeve J., Einecke G., Sis B., Hidalgo L.G., Famulski K., Matas A., Halloran P.F. (2012). Understanding the causes of kidney transplant failure: The dominant role of antibody-mediated rejection and nonadherence. Am. J. Transplant..

[27] Aubert O., Loupy A., Hidalgo L., van Duong Huyen J.-P., Higgins S., Viglietti D., Jouven X., Glotz D., Legendre C., Lefaucheur C. (2017). Antibody-Mediated Rejection Due to Preexisting versus De Novo Donor-Specific Antibodies in Kidney Allograft Recipients. J. Am. Soc. Nephrol..

[28] Zecher D., Tieken I., Wadewitz J., Zeman F., Rahmel A., Banas B. (2023). Regional Differences in Waiting Times for Kidney Transplantation in Germany. Dtsch. Ärzteblatt Int..

[29] Boerstra B.A., Boenink R., Astley M.E., Bonthuis M., Abd ElHafeez S., Arribas Monzón F., Åsberg A., Beckerman P., Bell S., Cases Amenós A. (2024). The ERA Registry Annual Report 2021: A summary. Clin. Kidney J..

[30] Bundesärztekammer (2021). Richtlinie gemäß § 16 Abs. 1 S. 1 Nrn. 2 u. 5 TPG für die Wartelistenführung und Organvermittlung zur Nierentransplantation. Dtsch. Ärzteblatt Online.

[31] de Ferrante H., Smeulders B., Tieken I., Heidt S., Haasnoot G.W., Claas F.H.J., Vogelaar S., Spieksma F. (2023). Immunized Patients Face Reduced Access to Transplantation in the Eurotransplant Kidney Allocation System. Transplantation.

[32] Kainz A., Kammer M., Reindl-Schwaighofer R., Strohmaier S., Petr V., Viklicky O., Abramowicz D., Naik M., Mayer G., Oberbauer R. (2022). Waiting Time for Second Kidney Transplantation and Mortality. Clin. J. Am. Soc. Nephrol..

[33] Heidt S., Witvliet M.D., Haasnoot G.W., Claas F.H.J. (2015). The 25th anniversary of the Eurotransplant Acceptable Mismatch program for highly sensitized patients. Transpl. Immunol..

