# Supplementary material for: A Novel Pre-Kidney Transplant Risk Score to Optimize Waiting List Management
Source: J Clin Med. 2026 Apr 16;15(8):3045. doi: 10.3390/jcm15083045 (PMC13117331; doi:10.3390/jcm15083045)
Supplement: Supplementary file 1 [file jcm-15-03045-s001.zip › Supplements_JCM_Revision1.pdf]

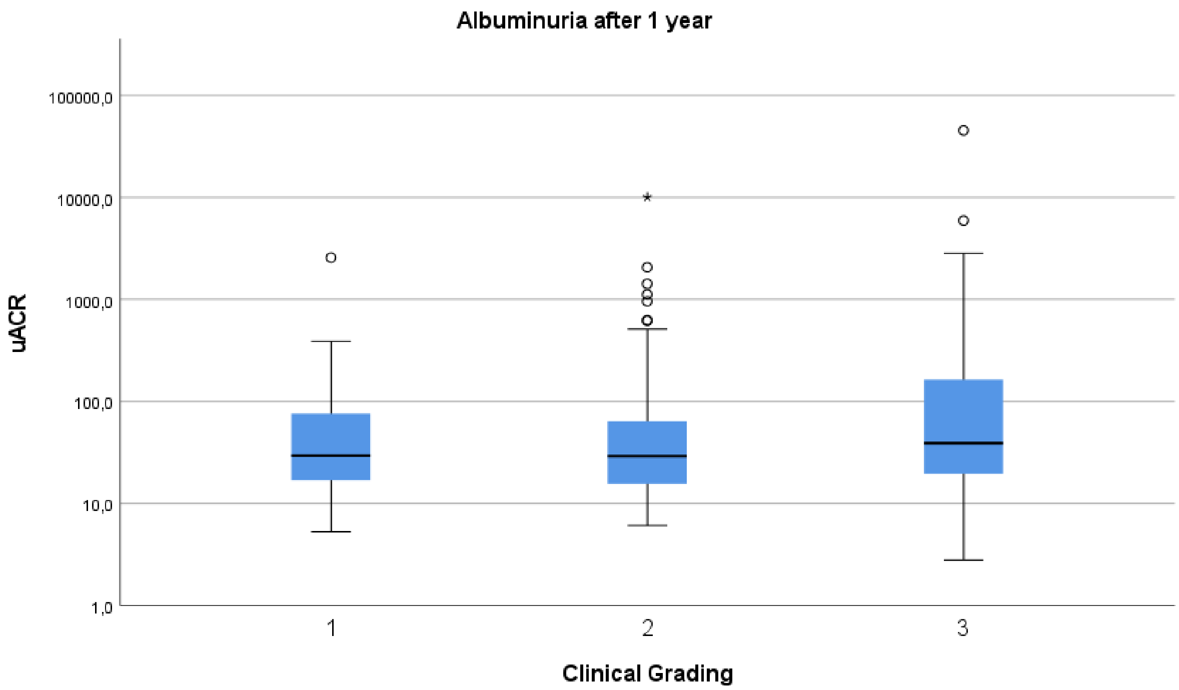

Supplementary Figure S1. Box plot: Comparison of uACR values at one year categorized by clinical grade (1: n = 61, 2: n = 179, 3: n = 144).

Graft Function after 1 year

Immunologic Grading	N	Mean	SD	p
Group A	299	45,6	20,4	
Group B	111	45,1	18,4	
Group C	31	40,4	17,1	
Total	441	45,1	19,7	0.29

Supplementary Table S1. Mean eGFR (CKD EPI) stratified by immunologic grading. Missing values for 25 patients (n = 441).

Albuminuria after 1 year

Immunologic Grading	N	Median	IQR	p
Group A	264	30.6	16.8 - 76.1	
Group B	92	35.6	15 - 65.8	
Group C	27	29.0	19.4 - 164.9	
Total	383	32.4	16.8 - 83	0.9

Supplementary Table S2. Mean uACR stratified by immunologic grading. Missing values for 82 patients (n = 383).

Biopsy proven rejections after 1 year by Clinical Grading

			No	Yes	Total	p
Clinical Grading	I	N	35	7	42	
		%	83.3%	16.7%	100.0%	
	II	N	144	30	174	
		%	82.8%	17.2%	100.0%	
	III	N	77	15	92	
		%	83.7%	16.3%	100.0%	
Total		N	256	52	308	
		%	83.1%	16.9%	100.0%	0.977

Supplementary Table S3. Number of rejections within 1 year after renal transplantation stratified by clinical grading. Only Muenster patients (n = 308).

Biopsy proven rejections after 1 year by Immunologic Grading

			No	Yes	Total	p
Immunologic Grading	A	N	172	30	202	
		%	85.1%	14.9%	100.0%	
	B	N	61	16	77	
		%	79.2%	20.8%	100.0%	
	C	N	22	5	27	
		%	81.5%	18.5%	100.0%	
Total		N	255	51	306	
		%	83.3%	16.7%	100.0%	0.493

Supplementary Table S4. Number of rejections within 1 year after renal transplantation stratified by immunologic grading. Only Muenster patients (n = 308).

Waiting time (days) by Clinical Grading

Clinical Grading	N Total	N ETKAS	Mean Total	Mean ETKAS	SD Total	SD ETKAS	Correlation Total	Correlation ETKAS
I	70	64	2,423	2,482	1,471	1,519		
II	206	176	2,613	2,713	1,161	1,164		
III	189	103	1,881	2,211	1,337	1,595		

Total	465	343	2,287	2,519	1,326	1,388	p < 0.001	p = 0.098

Supplementary Table S5. Mean waiting time in days stratified by clinical grading for all patients and patients receiving transplants through the ETKAS program (n = 465).

Waiting time (days) by Immunologic Grading

Immunologic Grading	N Total	N ETKAS	Mean Total	Mean ETKAS	SD Total	SD ETKAS	Correlation Total	Correlation ETKAS
A	314	226	2,099	2,346	1,362	1464		
B	114	97	2,707	2,852	1,218	1193		
C	35	19	2,622	2,915	980	1014		
Total	463	342	2,288	2,521	1,329	1389	p < 0.001	p = 0.002

Supplementary Table S6. Mean waiting time in days stratified by immunologic grading for all patients and patients receiving transplants through the ETKAS program (n = 465).

Listing time (days) by Clinical Grading

Clinical Grading	N	Mean	SD	p
I	66	564	774	
II	206	837	918	
III	184	715	872	
Total	456	748	883	0.007

Supplementary Table S7. Mean listing time (evaluation-to-listing interval) stratified by clinical grading. Missing values for 9 patients (n = 456).

Listing time (days) by Immunologic Grading

Immunologic Grading	N	Mean	SD	p
A	306	764	891	
B	113	702	850	
C	35	742	944	
Total	454	747	883	0.919

Supplementary Table S8. Mean listing time (evaluation-to-listing interval) in days stratified by immunologic grading. Missing values for 11 patients (n = 454).

NT days by Clinical Grading

Clinical Grading	N Total	N ETKAS	Median Total	Median ETKAS	IQR Total	IQR ETKAS	p Total	p ETKAS
1	42	36	256	277	40 - 664	34 - 655		
2	174	147	229	262	3 - 548	14 - 577		
3	92	20	157	384	0 - 460	13 - 1020		
Total	308	203	191	273	0 - 538	21 - 627	0.069	0.578

Supplementary Table S9. Number of NT days stratified by Clinical grading for all patients and patients receiving transplants through the ETKAS program. Only Münster patients (n = 308).

NT days by Immunologic Grading

Immunologic Grading	N	Median	IQR	p
A	202	151	0	
B	77	315	64	
C	27	349	161	
Total	306	191	0 - 538	<0.001

Supplementary Table S10. Number of NT days stratified by Immunologic grading. Missing values for 2 patients. Only Münster patients (n = 306).

Donor characteristics

Association (p), Correlation (p)	Donor Age	Donor Sex	Cold ischemia time	Mismatches
Mortality	0.209	0.569	0.793	0.141
Mortality + Graft Loss	0.435	0.217	0.891	0.121
eGFR	< 0.001, < 0.001	0.064, 0.037	0.037, 0.352	0.937, 0.004
uACR	0.966, 0.951	0.393, 0.299	0.177, 0.144	0.578, 0.670
Rejections	0.108, 0.084	0.112, 0.308	0.481, 0.793	0.950, 0.793

Supplementary Table S11. Mortality, Mortality and Graft Loss, eGFR, uACR and Rejections stratified by Donor Characteristics regarding association and correlation (only eGFR, uACR, Rejections). n = 460 (missing data in 5 patients) for Mortality, Mortality + Graft Loss, eGFR and uACR. Only Muenster patients (n = 308) for Rejections.

Interaction of Donor Age with Clinical Grading and eGFR

Association with eGFR	Before	After Mediator analysis
Clinical Grading	< 0.001	0.061
Donor Age	< 0.001	< 0.001
Interaction		0.281

Supplementary Table S12. Mediator analysis of the interaction of donor age with clinical grading and eGFR 1 year after Transplantation. n= 460 (missing data in 5 patients).

Charlson Comorbidity Index

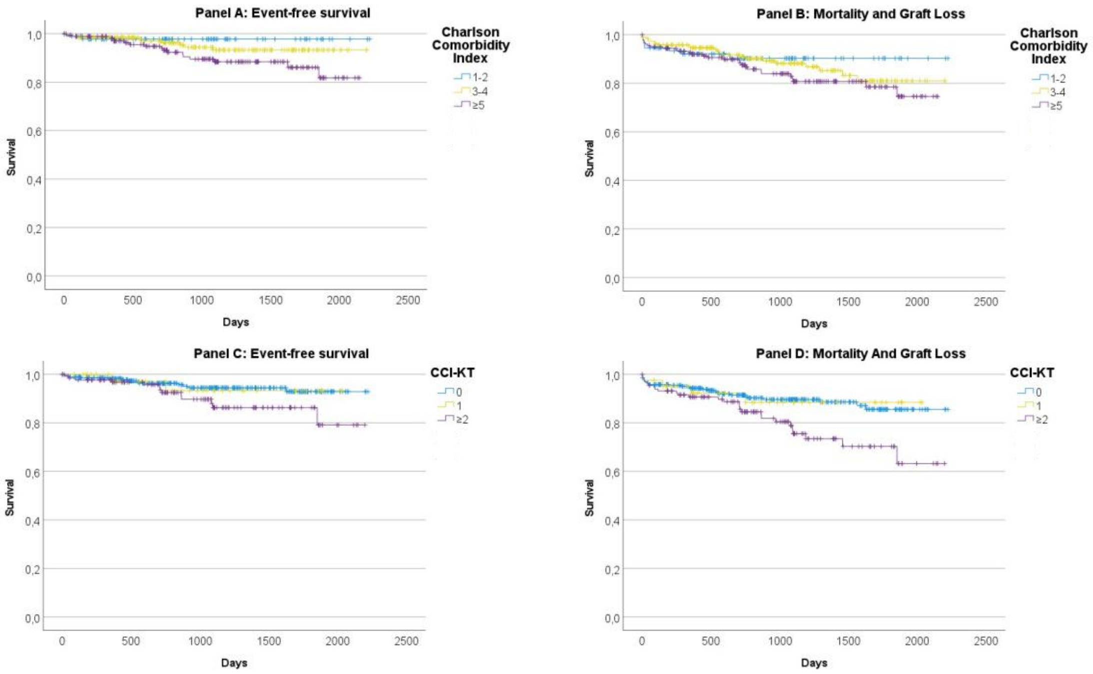

Supplementary Figure S2. Kaplan-Meier of Event-free survival categorized by CCI (Panel A) and CCI-KT (Panel C), mortality or graft loss categorized by CCI (Panel B) and CCI-KT (Panel D). n = 465.

Graft Function after 1 year

CCI	N	Mean	SD	p
1-2	88	56.8	19.1	
3-4	183	45.6	19.8	
≥5	172	38.6	17.0	
Total	443	45.1	19.7	<0.001

CCI-KT	N	Mean	SD	p

0	274	47.1	18.4	
1	41	46.4	25.3	
2	128	40.6	19.9	
Total	443	45.1	19.7	0.004

Supplementary Table S13. Mean eGFR (CKD EPI) stratified by Charlson Comorbidity Index and Charlson Comorbidity Index-KT.

Albuminuria after 1 year

CCI	N	Median	IQR	p
1	79	23	14 – 65	
2	156	29	16 – 82	
3	149	44	22 - 150	
Total	384	33	17 - 85	<0.001

CCI-KT	N	Median	IQR	p
0	241	28	16 – 73	
1	36	46	20 – 190	
2	107	42	21 – 118	
Total	384	33	17 - 85	0.012

Supplementary Table S14. Mean uACR stratified by Charlson Comorbidity Index and Charlson Comorbidity Index-KT.

Karnofsky Index, RCRI, MICA

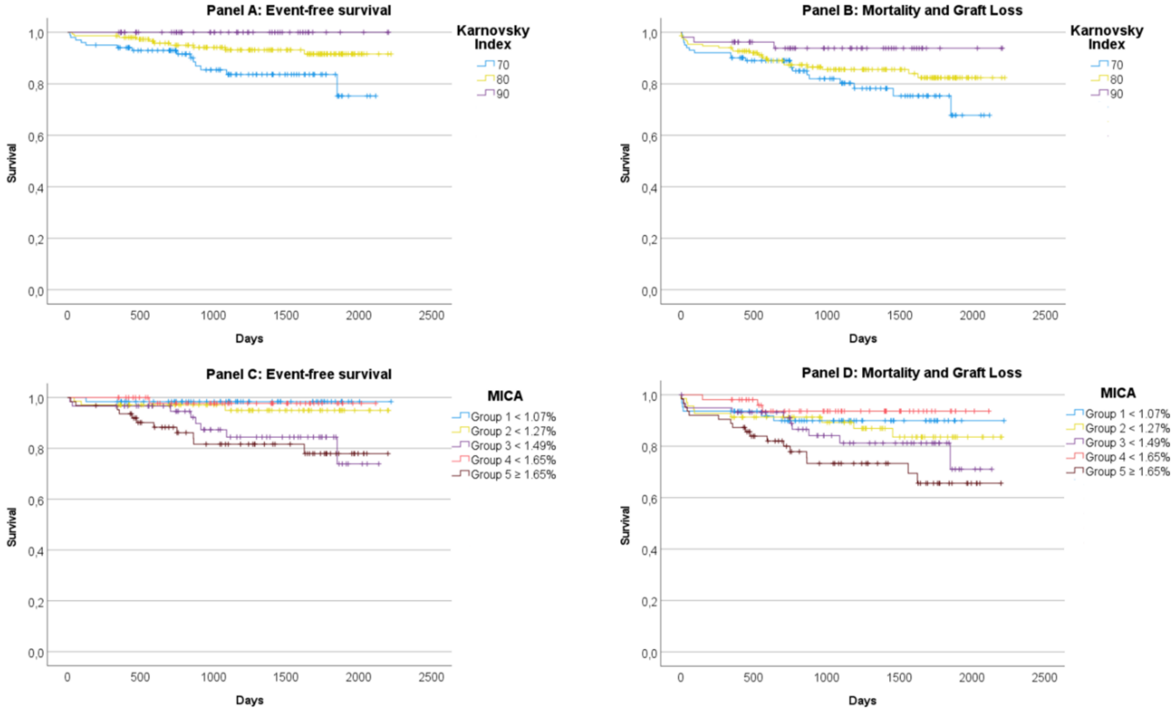

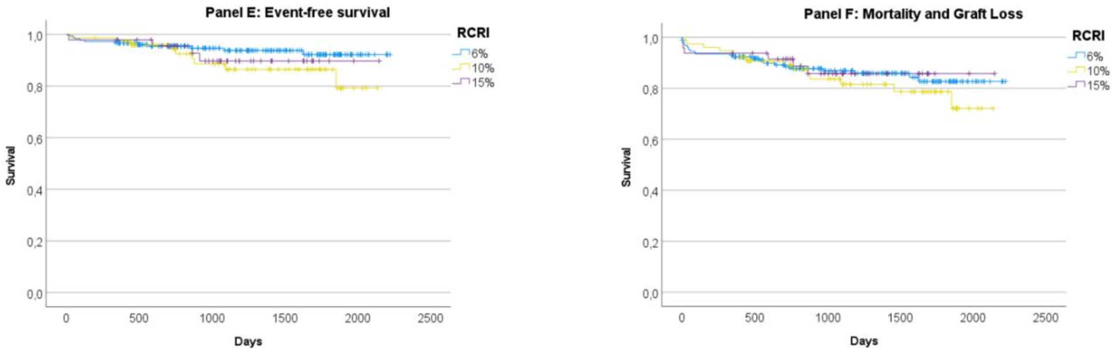

Supplementary Figure S3. Kaplan-Meier of Event-free survival categorized by Karnofsky Index (Panel A), MICA (Panel C) and RCRI (Panel E), mortality or graft loss categorized by Karnofsky Index (Panel B), MICA (Panel D) and RCRI (Panel F). n = 465.

Graft Function after 1 year

Karnofsky Index	N	Mean	SD	p
90	52	50.54	22	
80	140	43.61	17.9	
70	92	42.28	18.9	
Total	284	44.45	19.2	0.046

MICA	N	Mean	SD	p
Group 1	60	53.71	23.3	

Group 2	64	47.29	17.2	
Group 3	55	44.91	18.6	
Group 4	51	38.27	16.6	
Group 5	56	36.73	13.6	
Total	286	45.1	19.1	<0.001

RCRI	N	Mean	SD	p
6%	169	45.63	19.4	
10%	72	42.89	15.4	
15%	45	42.86	23.1	
Total	286	44.5	19.1	0.298

Supplementary Table S15. Mean eGFR (CKD EPI) stratified by Karnofsky Index, MICA (divided into 5 equal groups: < 1.07%, < 1.27%, < 1.49%, < 1.65% and ≥ 1.65%) and RCRI.

Albuminuria after 1 year

Karnofsky Index	N	Median	IQR	p
90	49	23.4	14 – 50	
80	135	32.4	17 – 80	
70	84	41.35	19 - 114	

Total	268	32.6	17 - 80	0.018

MICA	N	Median	IQR	p
Group 1	56	19.4	13 – 58	
Group 2	61	28.7	15 – 53	
Group 3	52	41.9	18 – 78	
Group 4	48	32.2	17 – 80	
Group 5	53	53.6	20 – 180	
Total	270	32.1	17 - 80	0.004

RCRI	N	Median	IQR	p
6%	161	25.2	16 – 65	
10%	68	44.05	23 – 110	
15%	41	44.8	17 - 87	
Total	270	32.1	17 - 80	0.008

Supplementary Table S16. Mean uACR stratified by Karnofsky Index, MICA (divided into 5 equal groups: < 1.07%, < 1.27%, < 1.49%, < 1.65% and ≥ 1.65%) and RCRI.

Head-to-head comparison of the scores

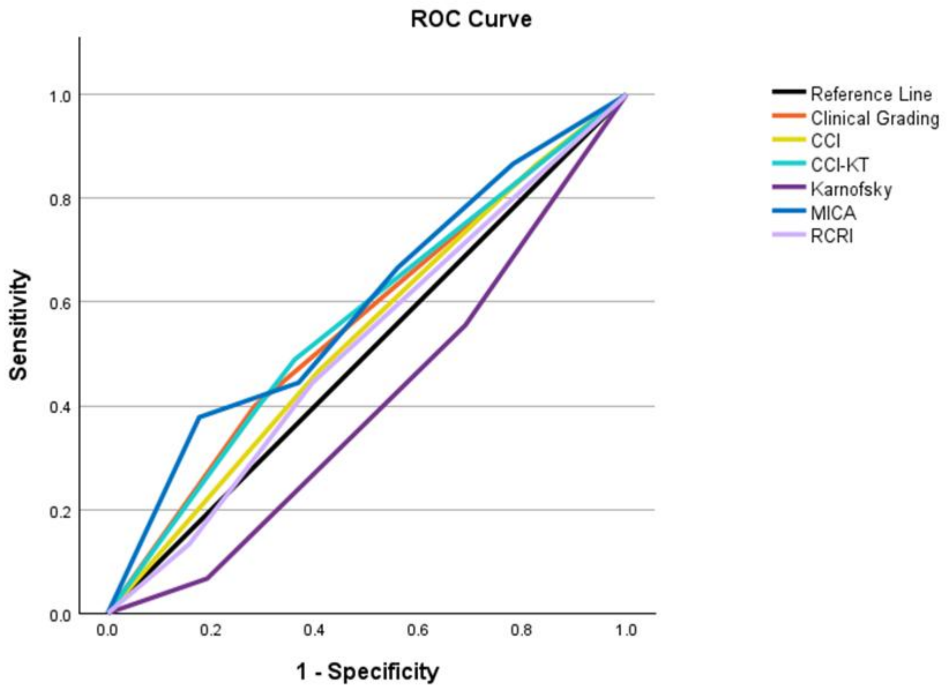

Supplementary Figure S4. ROC curves of the composite endpoint (mortality or graft loss) from the Clinical Grading (orange), Charlson Comorbidity Index (CCI, yellow), CCI-KT (green), Karnofsky Index (purple), MICA (divided into 5 equal groups: < 1.07%, < 1.27%, < 1.49%, < 1.65% and ≥ 1.65%, blue) and RCRI (light purple). n = 308.

Score head-to-head comparison

Score	AUC	95% CI	AUC diff.	p
Clinical Grading	0.560	0.476 – 0.644		
CCI	0.536	0.452 – 0.619	0.024	0.520
CCI-KT	0.565	0.484 – 0.646	- 0.005	0.940
Karnofsky Index	0.405	0.327 – 0.482	0.155	0.010
MICA	0.591	0.499 – 0.683	-0.031	0.229
RCRI	0.517	0.438 – 0.597	0.043	0.409

Supplementary Table S17. Calculated AUC from ROC analysis (Figure S4) with 95% confidence intervals. Difference in AUC and statistical significance (p) between the Charlson Comorbidity Index (CCI), CCI-KT, Karnofsky Index, MICA (divided into 5

equal groups: < 1.07%, < 1.27%, < 1.49%, < 1.65% and ≥ 1.65%), RCRI to clinical grading determined by non-overlapping CIs. n = 308.

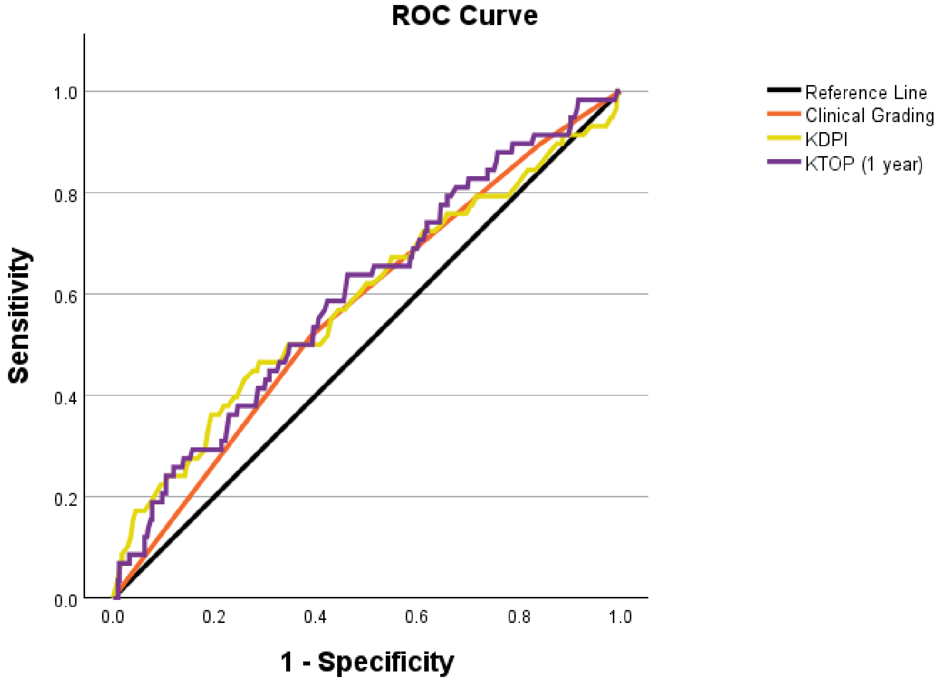

Supplementary Figure S5. ROC curves of the composite endpoint (mortality or graft loss) from the Clinical Grading (orange), KDPI (only graft failure, yellow) and KTOP (after 1 year, purple). n = 460 (missing data in 5 patients).

Score	AUC	95% CI	AUC diff.	p
Clinical Grading	0.570	0.498 – 0.641		
KTOP 1 year	0.599	0.520 – 0.678	-0.029	0.456
KDPI	0.587	0.501 – 0.673	- 0.017	0.695

Supplementary Table S18. Calculated AUC from ROC analysis (Figure S4) with 95% confidence intervals. Difference in AUC and statistical significance (p) between KTOP (1 year after RTx) and KDPI (only graft failure) to clinical grading determined by non-overlapping CIs. n = 460 (missing data in 5 patients).

Operational examples

Clinical Grading	Patient Profile	Re-Evaluation	Key Actions
I (low)	- 45 years<br>- only arterial hypertension	12 months	- Routine monitoring
II (intermediate)	- 62 years<br>- prior myocardial infarction,<br>- 18 months dialysis	6 months	- Frailty assessment,<br>- cardiovascular risk optimization<br>- non-invasive Ischemia testing
III (high)	- 74 years<br>- Diabetes mellitus<br>- Peripheral arterial disease<br>- Left ventricular ejection fraction 35%	3 months	- Multidisciplinary reviews<br>- Prehabilitation<br>- ESP/AM planning

Supplementary Table S19. Real-world operational examples for each risk level according to clinical grading.
